# Red to Far-Red Light Ratio Modulates Hormonal and Genetic Control of Axillary bud Outgrowth in Chrysanthemum (*Dendranthema grandiflorum* ‘Jinba’)

**DOI:** 10.3390/ijms19061590

**Published:** 2018-05-28

**Authors:** Cunquan Yuan, Sagheer Ahmad, Tangren Cheng, Jia Wang, Huitang Pan, Liangjun Zhao, Qixiang Zhang

**Affiliations:** 1 Beijing Key Laboratory of Ornamental Plants Germplasm Innovation & Molecular Breeding, National Engineering Research Center for Floriculture, Beijing Laboratory of Urban and Rural Ecological Environment, Key Laboratory of Genetics and Breeding in Forest Trees and Ornamental Plants of Ministry of Education, School of Landscape Architecture, Beijing Forestry University, Beijing 100083, China; yuancunquan@163.com (C.Y.); sagheerhortii@gmail.com (S.A.); chengtangren@163.com (T.C.); wangjia8248@163.com (J.W.); htpan2000@163.com (H.P.); 2 Department of Ornamental Horticulture, China Agricultural University, Beijing 100193, China; zhaolj5073@163.com

**Keywords:** chrysanthemum, R:FR, bud outgrowth, hormone, sucrose

## Abstract

Single-flower cut Chrysanthemum (*Dendranthema grandiflorum* ‘Jinba’) holds a unique status in global floriculture industry. However, the extensive axillary bud outgrowth presents a major drawback. Shade is an environment cue that inhibits shoot branching. Present study was aimed at investigating the effect of ratio of red to far-red light (R:FR) in regulating the lateral bud outgrowth of Chrysanthemum and the detailed mechanism. Results showed that the fate of axillary buds at specific positions in stem exhibited difference in response to R:FR. Decreasing R:FR resulted in elevation of abscisic acid (ABA) accumulation in axillary buds. Expression of ABA, indole-3-acetic acid (IAA) and strigolactones (SL) -related metabolism and signal transduction genes was significantly changed in response to low R:FR. In addition, low R:FR caused the re-distribution of sucrose across the whole plant, driving more sucrose towards bottom buds. Our results indicate that low R:FR not always inhibits bud outgrowth, rather its influence depends on the bud position in the stem. ABA, SL and auxin pathways were involved in the process. Interestingly, sucrose also appears to be involved in the process which is necessary to pay attention in the further studies. The present study also lays the foundation for developing methods to regulate axillary bud outgrowth in Chrysanthemum.

## 1. Introduction

Escalation and development of lateral branches are the major determinants of diverse shoot architectures, and this plastic trait is controlled by genetic and environmental regulators, and the interactive effect of both. Leaf axils nurture axillary meristems to produce axillary buds, which can then either remain arrested or transform into branches, creating plentiful architectural patterns [1]. In floriculture crops, axillary bud outgrowth impacts flower productivity and market value, and therefore has been considered a precious attribute in domestication and is one of the most targeted aspects by flower breeders when creating novelty. However, single-flower cut Chrysanthemum (*Dendranthema grandiflorum* ‘Jinba’) needs axillary buds remain arrested, letting only single stalk to grow vigorously with one flower at top. But it was backfire, most of the cultivated varieties produce number of axillary branches that need to manual disbudding timely during the development. Hence, how to control axillary bud outgrowth is an urgent problem that needs to be solved in cut Chrysanthemum.

In *Arabidopsis thaliana* L., the transformation of quasi-dormant axillary buds to active growth form is triggered by many intrinsic and extrinsic factors [2,3,4]. In *A. thaliana* grown under long days, upper buds start to elongate earlier, while buds in bottom leaf axils show a delay to elongate [5]. This gives the idea that bottom bud is variable in its growth kinetics and is contextually regulated by environmental factors, developmentally originated signals, or the co-influence of both [6,7,8,9]. Light signals with competitive environment show inhibitory effect on branching in many monocots and eudicots [6,10,11,12]. The low ratio of red to far-red light (R:FR) produced in the competitive environments is perceived by photoreceptors called phytochromes, with phytochrome B (phyB) being the major receptor. Signals sensed by phyB induce a series of adaptive responses called shade avoidance syndrome (SAS), bearing reduced shoot branching [13]. Profusion of indole-3-acetic acid (IAA), a natural auxin, mounts rapidly in infantile seedling of *A. thaliana* in response to low R:FR, contributing to SAS [14,15,16]. Thus, phyB deficiency and low R:FR are considered to be the major inhibitors of branching in *A. thaliana* by influencing the genetic expressions and pathways relating to bud kinetics [6,7,11,17]. 

Since antiquity, the axillary bud outgrowth through hormonal pathways has been an attention-grabbing field to study. Auxin has been admitted as systemic controller of axillary bud growth [1]. Basipetal auxin movement, through polar auxin transport, restricts bud outgrowth indirectly, not entering the lateral buds. This inhibitory effect of upper shoots on lower branches is a type of correlative inhibition called apical dominance. This correlative hindrance can be envisaged as the inhibitory effect from apical organs having more affinity to auxin source, though this may not be the absolute mechanism [18,19]. The precise mechanism of auxin mediated control of bud growth remains poorly defined although appealing models have been put forward. One of the models, the second messenger model, suggests auxin as regulator of second messengers like cytokinin and strigolactones, pushing them move into the bud and control bud outgrowth [20,21,22]. Another model proposes that axillary buds compete for transport capacity of auxin in the main stem [22,23,24,25]. In *A. thaliana* phyB deficiency promoted shade avoidance syndrome with inflated apical dominance, suggesting elevated auxin levels and pronounced signaling in the main stem [7]. Recently, less significance has been agreed to the function of auxin; rather, the association of sugar has been studied during apical dominance [26]. In fact, Morris et al. [19] argued that post-decapitation pea plant exhibited initiation of bud outgrowth before the auxin content changes in the adjacent stem tissues. It is an admitted fact that bud outgrowth innervates plenty of sugar metabolic activities and sugar transportation within buds [27,28,29,30,31,32].

Many researchers have coordinated the inhibition of branching with ABA abundance in axillary buds [33,34,35,36]. Some see this inhibition in context of ABA responses against R:FR [37,38]. It has been proposed that exogenous application of ABA causes side bud inhibition in may plants [39,40], while fluridone, an ABA inhibitor, promotes branching in *Rosa hybrida* L. [41]. Similarly, genetically altered Poplar with diminished sensitivity against ABA showed enhanced shooting [42]. According to Ortiz-Morea et al. [43], bud outgrowth in *Saccharum officinarum* L. has been linked with decreased ABA abundance in buds. Moreover, phyB deficiency resulted in retarded bud growth in *Sorghum bicolor* L. as compared to wild type, and overexpression of gene related to ABA [44]. *A. thaliana* exposure first to elevated R:FR and then to low R:FR suppressed stooling and elated ABA signaling [17]. Contrary to this exposing *A. thaliana* to low R:FR first restricted axillary buds and then subsequent exposure to high R:FR released the lateral buds [8]. Thus, increased R:FR suppressed the ABA signals and vice versa. A mutant deficient in ABA synthesis showed incomplete axillary bud suppression even in a low R:FR [8]. Now the ABA has been considered the inhibitor of bottom buds under both low and high red to far-red light ratios and may also suppress the cell cycle associated genes expression [45]. Expression of genes involved in independent auxin pathway of bud control are also inhibited by ABA, thereby inhibiting the IAA accumulation in the buds [45]. 

Describing the mechanism by which SL participates in the phyB-mediated R:FR changes Tao et al. [16] argues that the auxin is modulating the SL synthesis. Under high R:FR conditions, the synthesis of SL may be inhibited by phyB, and under low R:FR conditions, this inhibition is released, SL content increases, promoting the shading response phenotype. Reddy et al. [9] further demonstrated that phyB further supports the above speculation by inhibiting auxin signaling to promote the *A. thaliana* branching phenotype.

Different pathways controlling axillary bud development, including hormones and sugars, have been proposed to be integrated by *BRANCHED 1 (BRC1)* or its orthologs [26,46,47,48,49,50,51], though ABA down regulates *BRC1* [45]. But ABA may not be targeted by *BRC1* because it also regulates the expression of plentiful cell cycle related genes not identified before as responding against ABA [17]. Further studies have shown that the degree of influence of the photochromic effect on the inhibition of branching requires the participation of functional *BRC1*, *BRC2*, *AXR1*, *MAX2* and *MAX4*, while *BRC1* and *BRC2* participate in the photosensitizer-mediated branch-regulation response through different signaling pathways [50], involving the phyB-mediated hypocotyl elongation process. In *A. thaliana*, *BRC1* expression levels in axillary buds were elevated under high-density planting conditions, whereas complete inhibition of lateral buds under high-density planting conditions required *BRC1* involvement [51]. Kebrom et al. [52] first proposed that the inhibition of phyB inhibits the expression of *SbTB1* gene and induces axillary bud growth. However, when the external environmental conditions affect phyB in the inactive state, the *SbTB1* gene expression level is increased, thereby promoting the growth of axillary buds [11,52]. The results of the transcriptome data show that a large number of genes related to the cell cycle are down-regulated after shading, and the down-regulated gene promoter region is rich in TCP binding sites. However, in the case of high R:FR, the *max2phyB* double mutant and *max4phyB* double mutant showed similar phenotype of SL isoforms similar to SL mutants, which in turn inhibited the phylogenetic phenotype of phyB. The results showed that the SL pathway may act downstream of the phyB-dependent response pathway and requires SL participation in response to change in R:FR ratio [6].

Although roles for bud-confined ABA and auxin signaling in the main stem have been exploited substantially in context of axillary bud outgrowth in response to R:FR, absolute understanding still demands further insights. Besides, the relative systemic auxin and bud ABA pathways have neither been ascertained nor have the potential interceptions. Furthermore, whether other pathway especially for sucrose was involved in regulating of bud outgrowth in response to R:FR still remains unknown. In Chrysanthemum, R:FR modification of bud growth kinetics have not yet been fully determined. In this study the Chrysanthemum bud growth responses to ratio of red to far-red light were defined in association with hormonal and genetic pathways. Furthermore, we determined the roles of ABA on inhibiting bud outgrowth by applying exogenous ABA and the inhibitor fluridone to single-stem Chrysanthemum axillary buds. Our results indicated that the fate of axillary buds at specific positions in stem exhibited different response to R:FR, low R:FR not always inhibit bud outgrowth but its influence depends on the bud position in the stem in Chrysanthemum. ABA, SL and auxin pathway were involved in the process. It was interesting to find that sucrose also appears to contribute to this biological process which is necessary to pay attention in the further studies. The present study also lays the foundation for using R:FR to regulate axillary bud outgrowth in Chrysanthemum.

## 2. Results

### 2.1. The Axillary Buds at Specific Positions in Stem Showed Different Response to R:FR

**Figure 1:**
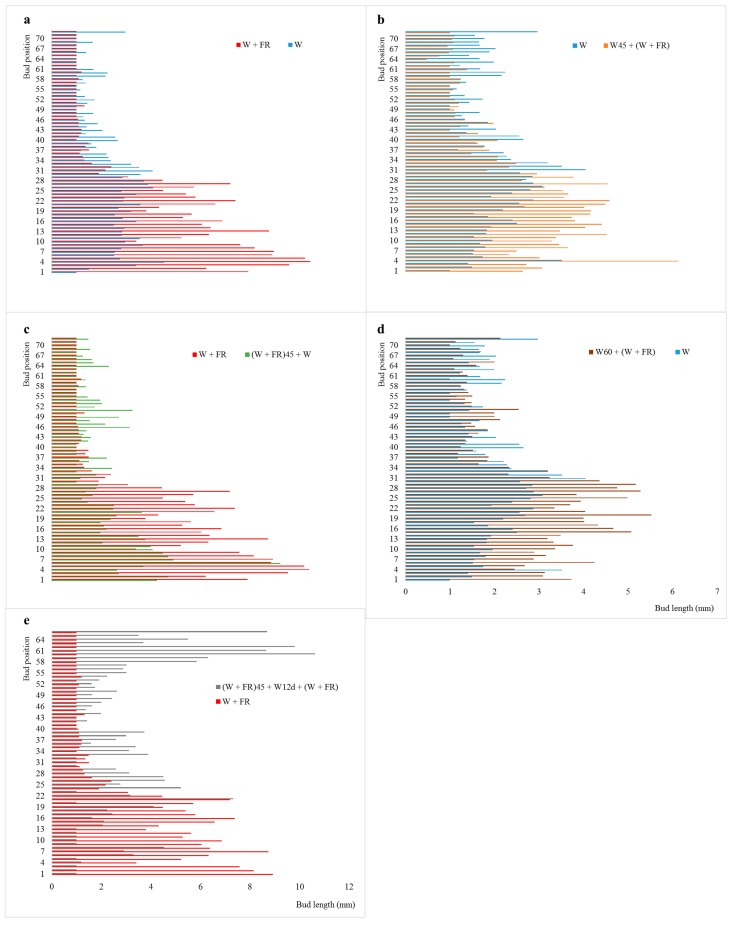

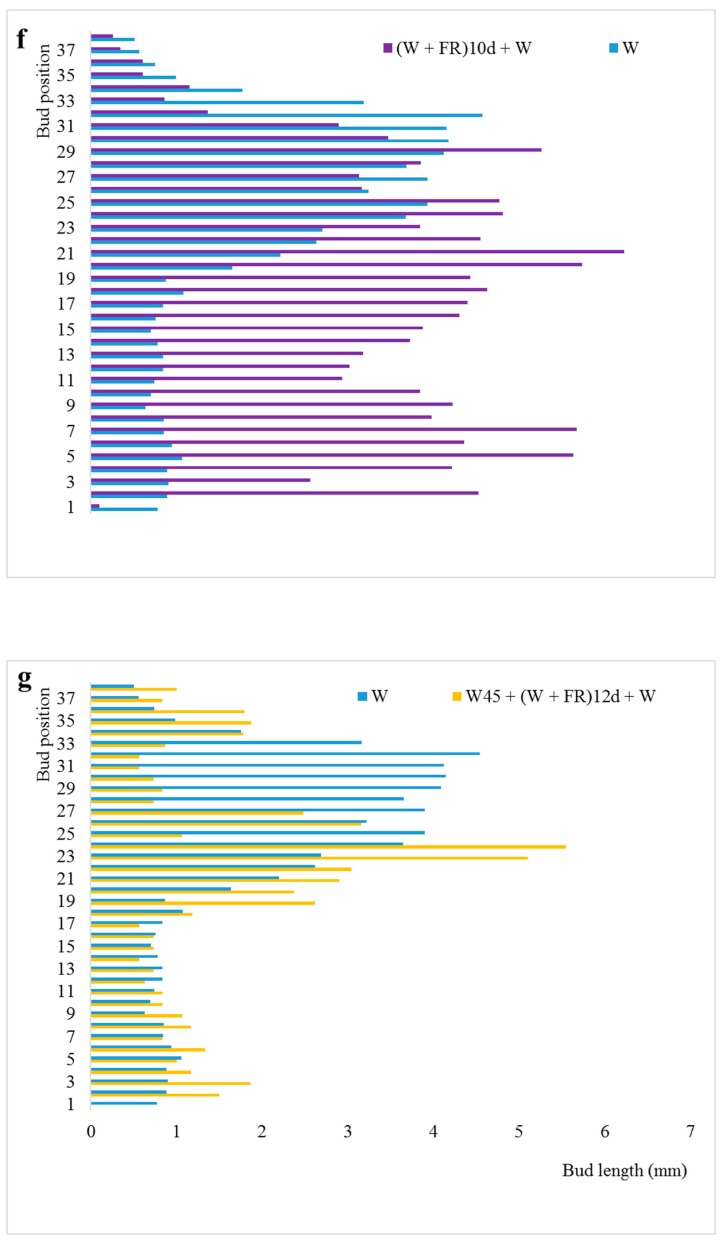
Axillary bud length under different ratio of red to far-red light (R:FR) treatments. (**a**–**e**) Bud length was measured 145 days after planting. (**f**,**g**) Bud length was measured 64 days after planting. (**a**) Plants were grown in low R:FR and high R:FR throughout the development stage; (**b**) Plants were first grown in high R:FR until 45 cm tall, and then either maintained under high R:FR or provided with low R:FR (High R:FR 45 + Low R:FR); (**c**) Plants were first grown in low R:FR until 45 cm tall, and then either maintained under low R:FR or provided with high R:FR (Low R:FR 45 + High R:FR); (**d**) Plants were first grown in high R:FR until 60 cm tall, and then either maintained under high R:FR or provided with low R:FR (High R:FR 60 + Low R:FR); (**e**) Plants were first grown in low R:FR until 45 cm tall, and then 1/2 plants were maintained in low R:FR and another 1/2 were provided with high R:FR for 12 days, then re-grown in low R:FR (Low R:FR 45 + High R:FR 12 days + Low R:FR); (**f**) Plants were first grown in low R:FR for 10 days at early seedling stage, and then grown in high R:FR (Low R:FR 10 days + High R:FR), plants that grown in high R:FR all the time were treated as a control; (**g**) Plants were first grown in high R:FR until 45 cm tall, and then 1/2 plants were maintained in high R:FR and another 1/2 provided with low R:FR for 12 days, then re-grown in high R:FR (High R:FR 45 + Low R:FR 12 days + High R:FR).

To fully understand the effect of R:FR on Chrysanthemum axillary bud outgrowth, 8 treatments including different development stages, different ways of treatment and different time duration were explored. In addition, to investigate whether axillary buds in different positions of stem respond to R:FR discrepantly, we examined the response of all the axillary buds from the first leaf axil to the last leaf axil in the stem. Results showed that axillary buds’ response is markedly different under changing light quality at different development stages. When the plants were grown in low R:FR and high R:FR throughout the development stage, separately, the upper bud length of plants that grown in low R:FR was much shorter than the plants grown in high R:FR. Contrarily, the lower bud length of plants that grown in low R:FR was much longer than plants grown in high R:FR (Figure 1a), suggesting that low R:FR inhibits upper bud outgrowth but promotes bottom bud outgrowth. When the plants were first grown in high R:FR until 45 cm height, standard height for axillary bud initiation in *D. grandiflorum* cv “*Jinba*” [47], then transferred into low R:FR, the upper bud length was much shorter than plants grown in high R:FR. Contrary to this, the bottom bud length was much longer than plants grown in high R:FR (Figure 1b). 

Plants first experiencing low R:FR until 45 cm height and then high R:FR, exhibited longer upper buds as compared to those still growing in low R:FR. However, the bottom bud length was much shorter than plants growing in low R:FR (Figure 1c). In the plants grown first in high R:FR until 60 cm height, then shifted to low R:FR, there was no difference in upper bud length, however, the bottom bud length was much longer than plants grown in high R:FR, but this effect was less strong (Figure 1d). After achieving 45 cm height in low R:FR, the plants shifting in high R:FR for 12 days, and then re-growing in low R:FR, produced longer upper buds compared with those still growing in low R:FR without any shifting. However, the bottom bud length was much shorter than plants grown in low R:FR (Figure 1e). Low R:FR treatment for 10 days at early seedling stage inhibited the upper bud outgrowth but largely promoted the bottom bud outgrowth (Figure 1f). 12 days low R:FR treatment at 45 cm height obviously inhibited the upper bud outgrowth, but for bottom bud, there was no difference (Figure 1g).

In short, early applying of low R:FR inhibited the upper bud outgrowth but promoted the bottom bud outgrowth in Chrysanthemum. When applying late in the development, after bud initiation, the inhibitory effect for upper buds was not obvious, but the facilitating effect for bottom buds was still exist, but was much weaker. The inhibitory effect on upper buds was not obvious for early stage for short time low R:FR exposure, however, the promotion effect was obvious for the bottom buds. Short time low R:FR treatment at the time of bud release inhibited the upper bud outgrowth, but the stimulatory effect was not obvious for bottom buds. These results suggested that low R:FR treatment not always inhibits the bud outgrowth but also promotes bud outgrowth, depending on the bud position in the stem. This is an interesting phenomenon distinct from the other species that low R:FR inhibits bud outgrowth. The divergent response of the axillary buds on R:FR suggests that the effect of R:FR on Chrysanthemum is comparatively complex.

### 2.2. R:FR Affect Differently the IAA, CK, GA and ABA Deposition in Buds

**Figure 2:**
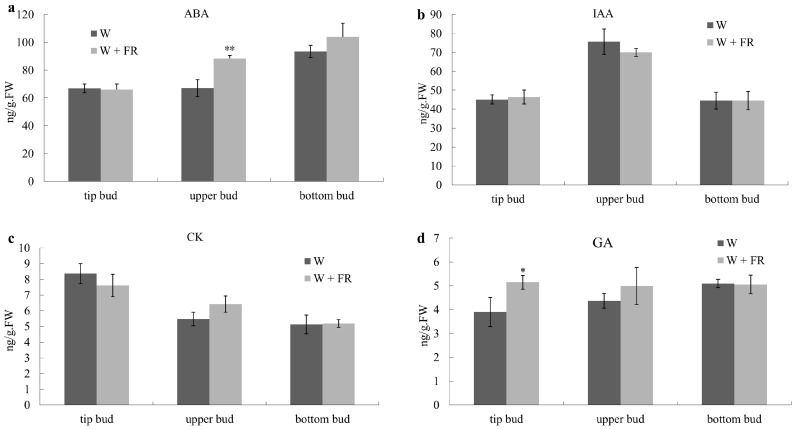
Abundances of ABA (**a**); IAA (**b**); CK (Cytokinin) (**c**) and GA3 (Gibberellin Acid 3) (**d**) in tip bud, upper bud and bottom bud of plants grown under high R:FR for 41 days (nearly 45 cm height) and then either maintained under high R:FR or provided with low R:FR for 12 h. FW, Fresh weight. Data are mean ±SE of three biological replicates. Asterisks indicate significant differences between the treatment conditions W and W + FR for each position-bud at *p* < 0.05 (*) and *p* < 0.01 (**) level.

The hormone levels were determined from 45 cm tall plants grown under High R:FR (W) continuously, or when they were transferred to Low R:FR (W + FR) for 12 h. The level of ABA in tip buds did not significantly change after treating with low R:FR, however, it elevated in axillary buds within 12 h under decreased R:FR, especially in upper buds. When compared with the upper buds and bottom buds, ABA level in bottom buds was much higher than in upper buds (Figure 2a). IAA content remained unaffected by R:FR in top and bottom buds (Figure 2b), however, the upper buds showed some response against control and simulated shading aided with far red light (W + FR). As for cytokinin (CK) content within the buds, the bottom bud was non-responsive against light variation, while those tip and upper buds showed contrasting behavior (Figure 2c). Tip bud was more responsive in white light as compared to upper buds, wherein more cytokinins accumulated in buds growing under simulated far-red light environment. GA content was significantly increased in tip buds but did not change significantly in axillary bud in response to low R:FR (Figure 2d). 

### 2.3. Sucrose Transport is Affected by Extended Application of R:FR

**Figure 3:**
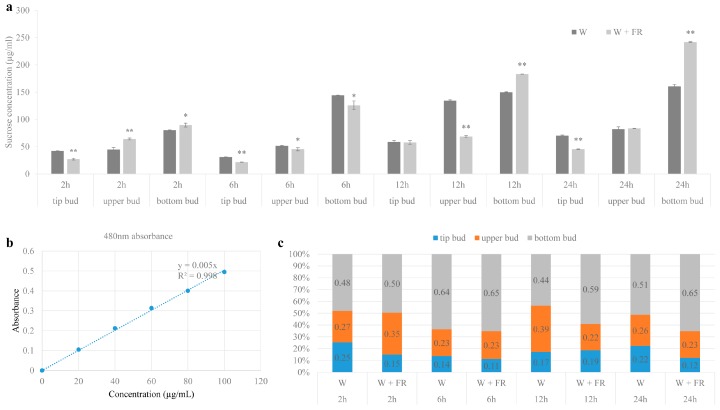
Sucrose accumulation in tip bud, upper bud and bottom bud of plants grown under high R:FR for 41 days (nearly 45 cm height) and then either maintained under high R:FR or provided with low R:FR for 2 h, 6 h, 12 h and 24 h. (**a**) Sucrose concentration; (**b**) Sucrose standardization curves, *p*-value = 8.075 × 10^−^^7^; (**c**) The distribution proportion of sucrose in tip bud, upper bud and bottom bud. Data are mean ±SE of three biological replicates. Asterisks indicate significant differences between the treatment conditions W and W + FR for each position-bud and each time-point at *p* < 0.05 (*) and *p* < 0.01 (**) level.

To investigate whether sucrose is involved in low R:FR-stimulated bud outgrowth, sucrose accumulation in tip bud, upper bud and bottom bud was examined after 2 h, 6 h, 12 h and 24 h of low R:FR treatment. As shown in Figure 3a, after 2 h of low R:FR application, sucrose content in tip bud was significantly decreased, instead, sucrose content in upper bud and bottom bud was significantly increased. After 12 h, sucrose content in tip bud was almost same in low R:FR (W + FR) compared with its control (W). However, sucrose content in upper bud was significantly decreased, contrarily, it was significantly increased in bottom bud. After 24 h, the sucrose accumulation in tip bud was also significantly decreased, while it had no change in upper bud, however, it was significantly increased in bottom bud (Figure 3a). Overall, sucrose accumulation was decreased in tip bud while increased in bottom bud with the time of low R:FR application. For upper bud, sucrose accumulation was increased in early time, and then began to decrease, and almost have no change after 24 h of low R:FR application (Figure 3a). Figure 3c shows the distribution proportion of sucrose in tip bud, upper bud and bottom bud, which can more intuitively reveal the sucrose transport and distribution at different positions of individual plant. Results showed that the sucrose distribution proportion in tip bud has been reduced continually with the time of low R:FR application. Meanwhile, the sucrose distribution proportion increased in both upper and bottom bud at early time. With the time of low R:FR application, the sucrose distribution proportion in upper bud also began to reduce at late time, but kept on increasing in bottom bud (Figure 3c). These results indicated that low R:FR caused the re-distribution of sucrose in whole plant of Chrysanthemum and drove more sucrose towards bottom buds, possibly providing a good sugar sink in the bottom plant parts.

### 2.4. The Expression of Max-Pathway Related Genes in Response to Low R:FR

**Figure 4:**
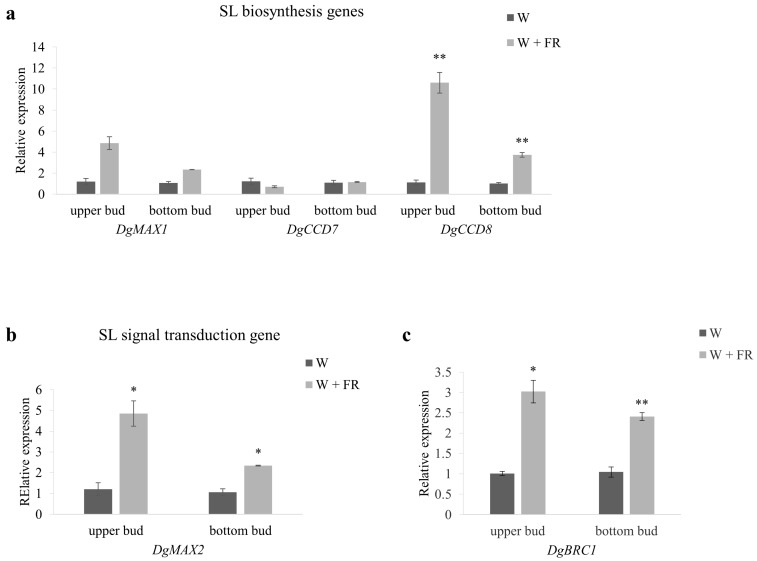
Expression profiles of SL-related genes in upper bud and bottom bud of plants grown under high R:FR for 41 days (nearly 45 cm height) and then either maintained under high R:FR or provided with low R:FR for 8 h. (**a**) SL biosynthesis *DgMAX1*, *DgCCD7* and *DgCCD8*; (**b**) SL signal transduction gene *DgMAX2*; (**c**) *DgBRC1*, a transcription factor that act as an integrator of branching signals within axillary buds and act downstream of SL pathway. Data are mean ±SE of three biological replicates. Asterisks indicate significant differences between the treatment conditions W and W + FR for each position-bud at *p* < 0.05 (*) and *p* < 0.01 (**) level.

To test the involvement of strigolactones in low R:FR-stimulated bud outgrowth, expression patterns of SL biosynthesis genes (*DgCCD7*, *DgCCD8*, *DgMAX1*) and signal transduction gene (*DgMAX2*), in upper and bottom buds, were examined in response to low R:FR (Figure 4). Among these genes, the expression of *DgCCD8* and *DgMAX2* were significantly up regulated after low R:FR treatment. In upper buds, *DgCCD8* and *DgMAX2* gene expression was elevated 9.47 and 4.03 times in the W + FR compared to W, separately. In bottom buds, *DgCCD8* gene expression was elevated 3.66 and 2.20 times in the W + FR compared to W, separately (Figure 4a,b). *DgMAX1* gene expression was also up regulated in response to low R:FR treatment (Figure 4a). 

*BRC1*, a gene coding for a transcription factor that acts as an integrator of branching signals within axillary buds to arrest bud outgrowth with expression well related to bud outgrowth. BRC1 acts downstream of the SL pathway and is modulated by strigolactones to control shoot branching [46,51]. Results showed that *DgBRC1* expressions were significantly up regulated in the W + FR compared to W both in upper and bottom buds, the expression patterns were similarly to *DgMAX2* (Figure 4b,c). These results indicated that max-pathway genes were involved in the regulation of axillary bud outgrowth in response to low R:FR in Chrysanthemum.

### 2.5. The Expression of ABA-Related Genes in Response to Low R:FR 

**Figure 5:**
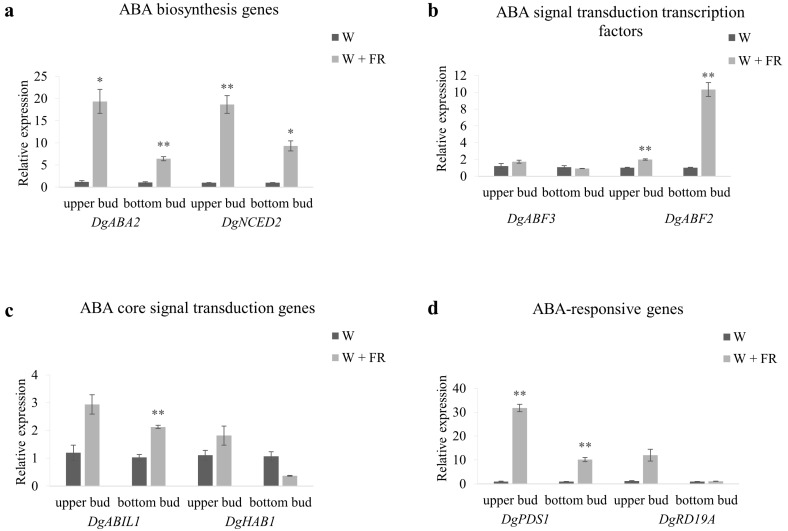
Expression profiles of ABA-related genes in upper bud and bottom bud of plants grown under high R:FR for 41 days (nearly 45 cm height) and then either maintained under high R:FR or provided with low R:FR for 8 h. (**a**) ABA biosynthesis genes *DgABA2* and *DgNCED2*; (**b**) ABA signal transduction transcription factors *DgABF3* and *DgABF2*; (**c**) ABA core signal transduction genes *DgABIL1* and *DgHAB1*; (**d**) ABA-responsive genes *DgPDS1* and *DgRD19A*. Data are mean ±SE of three biological replicates. Asterisks indicate significant differences between the treatment conditions W and W + FR for each position-bud at *p* < 0.05 (*) and *p* < 0.01 (**) level.

The accumulation of ABA in axillary buds after low R:FR treatment suggested that ABA might be involved in bud response to low R:FR. To further test this, the expression of ABA-related genes including biosynthesis, signal transduction and responsive genes were examined. Decreasing R:FR resulted in a significant up regulation of ABA biosynthesis genes’ expression (Figure 5a). The expression levels of ABA signal transduction transcription factor, *DgABF2*, and ABA core signal transduction gene, *DgABIL1*, were also significantly higher than that in control (Figure 5b,c). The transcript abundance of ABA responsive gene, *DgPDS1*, was much higher in low R:FR compared with the control in high R:FR (Figure 5d). The expression patterns of *DgABA2*, *DgNCED2*, *DgABF2*, *DgABIL1* and *DgPDS1* were similar. However, when compared with the upper bud and bottom bud, the expression changes were much stronger in upper bud than in bottom bud in response to low R:FR. In summary, low R:FR resulted in the increased expressions of both ABA biosynthesis and signal transduction genes, thus providing the support for ABA involvement in bud outgrowth regulation in response to R:FR.

### 2.6. The Expression of Auxin-Pathway Related Genes in Response to Low R:FR

**Figure 6:**
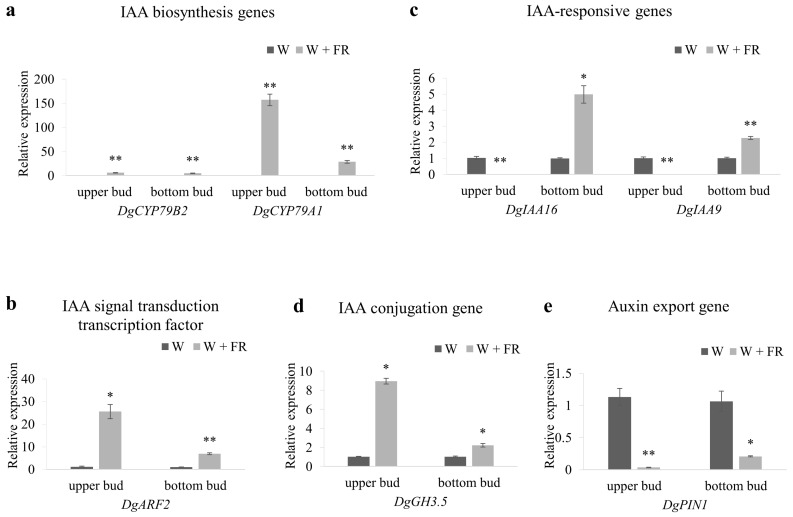
Expression profiles of IAA-related genes and auxin export gene in upper bud and bottom bud of plants grown under high R:FR for 41 days (nearly 45 cm height) and then either maintained under high R:FR or provided with low R:FR for 8 h. (**a**) IAA biosynthesis genes *DgCYP79B2* and *DgCYP79A1*; (**b**) IAA signal transduction transcription factor *DgARF2*; (**c**) IAA-responsive genes *DgIAA16* and *DgIAA9*; (**d**) IAA conjugation gene *DgGH3.5*; (**e**) Auxin export gene *DgPIN1*. Data are mean ±SE of three biological replicates. Asterisks indicate significant differences between the treatment conditions W and W + FR for each position-bud at *p* < 0.05 (*) and *p* < 0.01 (**) level.

To understand response of the auxin pathway genes to R:FR in axillary buds of Chrysanthemum, expression analysis of IAA biosynthesis, conjugation, and signal transduction transcription factors and responsive genes, in upper and bottom buds, was performed under low R:FR (Figure 6). Results showed that expression of IAA biosynthesis genes were significantly up regulated in low R:FR compared with high R:FR. The expression of *DgCYP79B2* and *DgCYP79A1* genes were increased 5.68 and 152.31 times in upper axillary buds and, 3.94 and 28.48 times in bottom buds respectively (Figure 6a). Transcript abundance of IAA signal transduction transcription factor was increased after low R:FR treatment (Figure 6b). Moreover, the expression level of IAA-conjugation gene *DgGH3.5*, was also significantly up-regulated with upper and bottom buds showing 11.9 and 2.2 times increase in transcript level, respectively (Figure 6d). However, IAA-responsive genes showed the opposite expression tendency in upper bud and bottom bud in response to low R:FR (Figure 6c). The expression levels of *DgIAA16* and *DgIAA9* were significantly decreased in upper bud but increased in bottom bud after low R:FR treatment (Figure 6c). Overall, expression changes of IAA-related gene were much stronger in upper bud than in bottom bud in response to low R:FR (Figure 6a–d).

PIN-FORMED1 (PIN1) proteins are known to be the key regulators of directional auxin transport and are the concern of bud outgrowth [2]. Results showed that reduction of the R:FR, achieved by far-red enrichment of a standard white-light background, caused a sharp decrease of *DgPIN1* expression (Figure 6e). 

### 2.7. Exogenous ABA Suppressed Axillary Bud Outgrowth 

**Figure 7:**
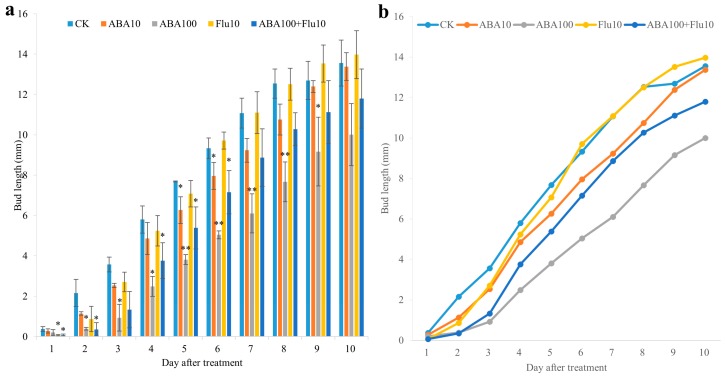
Exogenous ABA suppressed axillary bud outgrowth. Signal-bud node was inserted into the split-plate with MS media supplied with DMSO (control, CK), 1 μm ABA, 10 μm ABA, 100 μm ABA, 10 μm ABA biosynthesis inhibitor fluridon, and 100 μm ABA + 10 μm fluridon, separately. Bud length was measured from 1 day to 10 days by using camera and ImageJ software. (**a**) The bud length in different treatments and their difference comparison; (**b**) Bud length changing trend in different treatment in a visual sweep way. Data are mean ±SE of three biological replicates. Asterisks indicate significant differences between each treatment with CK for each time point at *p* < 0.05 (*) and *p* < 0.01 (**) level.

Auxin, strigolactones, cytokinins and gibberellins have been demonstrated to play roles in regulation of Chrysanthemum bud outgrowth [47,53,54,55,56,57]. However, we don’t know whether ABA can regulate Chrysanthemum bud outgrowth. To analyze the function of ABA in regulating bud outgrowth in Chrysanthemum, a split bud outgrowth assay supplemented with dimethyl sulfoxide (DMSO), 1 μm, 10 μm, 100 μm ABA and 10 μm ABA biosynthesis inhibitor fluridon and 100 μm ABA + 10 μm fluridon was conducted, separately. This assay have been used in previous studies to examine the effect of exogenous hormones on bud outgrowth in Chrysanthemum [47]. In this assay ABA was supplied to both the apical and basal end of a single bud. Results showed that axillary bud outgrowth rate was much slower in the medium supplied with ABA compared with medium supplied with DMSO. Bud length was significantly shorter in median supplied with 100 μm ABA compared with medium supplied with DMSO, in addition, supplying 10 μm fluridon and 100 μm ABA together weakened this inhibitory effect to some extent, suggesting the inhibitory effect of ABA on axillary bud outgrowth in Chrysanthemum (Figure 7a,b).

## 3. Discussion

### 3.1. The Axillary Bud in Different Positions of Plant Showed Varied Response to R:FR Change

Plant branches are regulated by the complex interactions between hormones, developmental and environmental factors. During development, plants can integrate environmental and endogenous hormonal signals and adjust branches to adapt to environmental changes. Light quality (the ratio of red to far red, R:FR) is an important environmental factor that regulates shoot branching, in which low R:FR often triggers shade avoidance syndrome (SAS) and is characterized by a reduced capacity for axillary buds to grow out. Low R:FR inhibited bud outgrowth has been reported in many species such as *A. thaliana* [6,7,17], *Brassica dilatatum* L. [58], *Lolium multiflorum* L. [59], *Rosa hybrida* L. [60] and *Trifolium repens* L. [12]. In this study, axillary buds in different positions showed varied response to R:FR change. When low R:FR treatment was conducted from an early seedling stage, the upper bud outgrowth was inhibited, on the contrary, the bottom bud outgrowth was promoted. Ten days of low R:FR treatment at early seedling stage also have the same effect (Figure 1a,f). When applying low R:FR treatment at the time of axillary bud release and outgrowth, the upper and bottom axillary bud showed different response, wherein the upper buds were inhibited and the bottom buds were promoted finally. Twelve days of high R:FR treatment at this time inhibited upper bud outgrowth but had negligible effect on bottom bud outgrowth (Figure 1b,g). Applying low R:FR treatment after axillary buds had a negligible effect on upper bud growth, but slightly promoted the bottom bud outgrowth (Figure 1d). In contrast, applying high R:FR to low R:FR conditions promoted the upper bud outgrowth, but inhibited the bottom bud outgrowth, 12 days of high R:FR treatment also have the same effect (Figure 1c,e). These results indicated that axillary buds respond differently to R:FR changes which depends on bud position and treatment time and way in Chrysanthemum. Different response of axillary buds to R:FR change had also been reported in *A. thaliana*, in which low R:FR inhibited the outgrowth of buds from lower positions but promoted the elongation of branches at upper positions [9]. This is completely different from our findings in Chrysanthemum. This may suggest that the effect of R:FR on plant shoot branching is comparatively complex. 

### 3.2. Hormones Were Involved in the Regulation of Axillary Bud Outgrowth in Response to Low R:FR

Bud growth regulation by R:FR signaling is far more complex and at least two routes govern this process. Initially, Reddy et al. [8] pointed out that elevated R:FR reduced the ABA concentration in buds, allowing low R:FR retarded buds to grow. Later on this idea was further supported by Gonzalez-Grandio et al. [17] whereby ABA related genes were shown to be responsive against R:FR. Subsequent studies by Reddy and Finlayson [9] demonstrated that branching suppression in a mutant with phyB-deficiency was caused by variations in auxin signaling in the stem. According to Holalu and Finlayson [1], axillary bud outgrowth was increased rapidly with increasing R:FR, and both ABA signaling and its abundance in buds were decreased due to low R:FR. Bud formation may be the central clue behind these changes as ABA was compulsory for restricting elongation of the bottom bud under low R:FR [8], and this idea was further strengthened by Yao and Finlayson [45], suggesting that buds were inhibited by exogenous application of ABA under high R:FR. Previous studies have also shown that ABA levels in axillary buds are positively correlated with degree of axillary bud dormancy [33,34,36]. In addition, low levels of red and far red light (Red: Far Red = R:FR, equivalent to shading) promote ABA accumulation, thereby inhibiting plant branching; high levels of red and far red (R:FR) reduced the accumulation of ABA content and promoted plant branching [37,38]. ABA or ABA inhibitors were used in plants such as *A. thaliana*, tomato, rose, pea, poplar and other plants to limit the axillary bud elongation [39,40,41,42]. But the role of ABA in controlling plant branching and its mechanism of regulating plant architecture have not yet been fully resolved.

Consistent with the previous findings, the ABA content in the upper axillary buds of Chrysanthemum was significantly increased, while a slight increase was observed in the bottom buds by decreasing the R:FR (Figure 2a). The expression of ABA biosynthesis gene *DgNCED2* was up-regulated 18.58-fold and 9.22-fold in the upper and bottom buds, respectively, after shading, and the expression level of *DgABA2* gene was up-regulated 16.19 and 5.98 times in the upper and bottom shoots respectively (Figure 5a). Since the ABA content was positively correlated with the degree of axillary bud dormancy, it was shown that the shading further promoted the dormancy of the axillary buds of Chrysanthemum, thus eventually inhibiting the elongation of axillary buds, especially for the upper bud. Further detection of ABA signal transduction and response gene expression revealed that a large number of ABA signal transduction-related and response gene expressions were significantly up-regulated in upper buds after shading, indicating that ABA sensitivity and response were enhanced in axillary buds after shading (Figure 5). For bottom buds, although the expression of ABA signal transduction-related genes *DgABF2* and *DgABIL1* were up regulated, the expression of *DgABF3* and *DgHAB1* were decreased, notably different from the upper buds. The changes of ABA content and ABA-related gene expression patterns in upper and bottom buds were basically in line with the morphological changes. To further study the function of ABA in regulation of bud outgrowth in Chrysanthemum, exogenous ABA and its inhibitor were used to treat with the single-bud node. We found that exogenous ABA inhibited the Chrysanthemum bud outgrowth, and its inhibitor can weaken this inhibitory effect (Figure 7a,b). From these findings, we believed that ABA was involved in shading-mediated inhibition of axillary buds of Chrysanthemum. However, our findings were much different from *A. thaliana*. In *A. thaliana*, low R:FR inhibited the growth of specific lower buds, and this could be inversed by increasing R:FR, the third bud from the top to the bottom was the most responsive to low to high R:FR [8]. ABA has been shown to inhibit lower bud growth under both high and low R:FRs [45]. These results indicate that the regulation process is complex and other pathways maybe also involved besides ABA. 

SL is considered to be involved in *A. thaliana* response to shade-induced axillary bud growth during the regulatory process [6], whereas *MAX2* is thought to participate in shading-induced hypocotyl elongation processes. In the present study, the expression level of *DgMAX2* gene in axillary buds of Chrysanthemum was significantly up-regulated in shade, which indicated that shade enhanced the SL signal in axillary buds of Chrysanthemum (Figure 4b). At the same time, lower R:FR caused the expression of *DgCCD8* and *DgMAX1* to up-regulate in the axillary buds (Figure 4a). Decreasing R:FR resulted in the increasing SL biosynthesis gene expressions, leading to elevated SL accumulation in the axillary buds, thereby suppressing the bud outgrowth. Up-regulation of SL signal gene *DgMAX2* after decreasing R:FR also suggested that the SL signal was strengthened. Considering that SL could directly act on axillary buds and inhibit their growth [57], shade could lead to the accumulation of SL in axillary buds of Chrysanthemum, the enhancement of SL signals and the growth of axillary buds. 

*BRC1* act as an integrator of branching signals within axillary buds to arrest bud outgrowth with expression well related to bud outgrowth. *BRC1* acts downstream of the SL pathway and is modulated by strigolactones to control shoot branching [46,51]. In our study, decreasing R:FR induced the significant up-regulation of *DgBRC1* in axillary buds, the expression patterns were similarly to *DgMAX2* (Figure 4b,c), indicating that the shade promotes the dormancy of axillary buds in Chrysanthemum. 

Auxin signaling plays an important role in regulating the growth of plant axillary buds. In *A. thaliana*, the *AXR1* gene is necessary in response to axillary bud growth regulation induced by the light signals [6]; in *A. thaliana*, phyB promotes branching by inhibiting auxin signaling [7]. The shade mainly works through the phyB pathway to control the branching. It is known that the auxin signal dependent on *AXR1* is usually converted to bud activity to control axillary bud growth [50]. In this study, decreased R:FR caused the significantly up-regulation of IAA-related biosynthesis and signal transduction genes (Figure 6), indicating the enhancement of auxin signaling and inhibition of bud outgrowth. When comparing with the upper and bottom buds, the enhancement of auxin signaling is much more obvious in upper buds, suggesting the more intensity of bud inhibition. Besides auxin signaling, auxin transport also plays important roles in the regulating of bud outgrowth [2]. According to the theory of channel hypothesis, the active state of axillary buds is positively correlated with the polar auxin transport (PAT) in the axillary buds. PIN-FORMED1 (PIN1) proteins are known to be the key regulators of directional auxin transport and its content was considered as directly related with the level of axillary bud auxin transport capacity. In *A. thaliana*, high R:FR promoted bud outgrowth accompanies a significantly elevated expression levels of *PIN1* in axillary buds under high R:FR [1]. In this study, the expression level of *DgPIN1* gene in the axillary buds of Chrysanthemum decreased in shade, which may forecast the reduced auxin output capacity in axillary buds and inhibition of the bud release as a result (Figure 6e). 

According to the above discussion, we hypothesized that hormonal pathway including SL pathway, ABA pathway and auxin pathway were involved in the regulation of axillary bud outgrowth in response to low R:FR in Chrysanthemum. 

### 3.3. Sucrose Maybe an Important Regulator of Shoot Branching and Bud in Response to the R:FR 

Sugar signaling is the integral regulator of plant growth and development during the whole life cycle. Sugar can start nutrient mobilization, cotyledon greening, hypocotyl elongation and shoot development during the plant germination [61,62,63,64,65,66,67,68,69]. Despite being the important source of energy and carbon, sugars also possess hormone-like roles as key messengers during signal transduction. The crucial role of sugar as a signaling agent is well explained by the plenty of sugar signaling and sensing mechanisms [70,71]. Photosynthetic production of sugar in plants is an essential process, and sugar coordinates and modulates environmental cues and the internal regulators to drive growth and development [68,72]. Although hexoses are considered the major signaling factors in plants, sucrose specific pathways also have pivotal roles in the regulation of plant development and gene expressions [73,74]. 

In the present study, sucrose accumulation in tip bud and axillary buds changed in response to R:FR. The sucrose accumulation in tip bud decreased continually with the time of low R:FR application. Meanwhile, the sucrose accumulation increased in both upper and bottom buds at an early time, but decreased in bottom bud at a late time and still increased in bottom buds. As a result of this, the sucrose accumulated in bottom buds. On the whole, the low R:FR caused the re-distribution of sucrose from shoot tip to lower position axillary buds, driving more sucrose towards bottom buds finally (Figure 3). From the sugar demand and hormone signaling model of shoot branching, sugar demand of the shoot tip and that limiting their availability from shoot tip to axillary buds is central to the maintenance of apical dominance. In this model, sugars are predominantly responsible for the initial bud release, while hormones are predominantly involved in prioritizing the later stage of bud outgrowth [49]. Except as a signaling molecule, sugars also play as main substrate for plant growth. Once a lateral bud has started to form a branch, sustained growth of this branch requires a steady supply of sugars [75]. It has been reported that treatments with sucrose in the medium resulted in stronger axillary bud growth in Chrysanthemum [53]. Based on this, we speculated that low R:FR caused the more sucrose accumulation in bottom buds and offered more possibility to bud release and outgrowth. Contrarily, decreased accumulation in tip and upper buds caused by low R:FR made it more likely to be inhibited. Therefore, sucrose maybe partially responsible for the upper bud inhibition and bottom bud promotion in response to low R:FR. Of course, we need more evidences to support our hypothesis in future. Although it has not been reported before that sucrose was involved in bud response to R:FR, our results suggested that sucrose maybe an important regulator of shoot branching in responses to R:FR and need to pay attention in the future studies. Of course, the detailed mechanism needs extensive research.

Although our study showed that hormones including ABA, SL and auxin were involved in bud response to R:FR, how is ABA integrated into the currently known plant branch control network still needs to study further. In addition, ABA may play a role in the process to axillary bud release, and sucrose also plays a key role in the process of axillary bud release, and whether there is a link between ABA and sucrose in regulating axillary bud growth especially in bud release, is a point worthy to ponder.

In conclusion, the integrated study of upper bud and bottom bud response to the R:FR demonstrates that axillary buds from different positions in the stem exhibited significant different morphological, physiological and molecular characteristics. Low R:FR not always inhibits bud outgrowth but its influence depends on the bud position in the stem in Chrysanthemum. Therefore, attention should be paid on bud position in further similar research. The accumulation of ABA content and change of ABA-related gene expression in axillary buds after low R:FR combined with exogenous ABA treatment suggested that ABA acts as a regulator of bud outgrowth in Chrysanthemum. This result further expanded the kinds of hormone in the regulation of bud outgrowth in Chrysanthemum. The significant changes of SL biosynthesis and signal transduction genes in axillary buds in response to low R:FR suggested that local changes in SL biosynthesis and signaling may play roles in regulating bud outgrowth in Chrysanthemum. The sucrose content in tip bud and axillary buds changed rapidly from 2 h in response to low R:FR treatment. Low R:FR caused the sucrose accumulated from the apical bud to bottom axillary buds. On the whole, low R:FR caused the re-distribution of sucrose from apical bud to lower position axillary bud and drove more sucrose towards bottom buds finally (Figure 3). These results made us to make an attractive speculation that sucrose maybe an important regulator of shoot branching and bud responses to the R:FR. 

Colligating above results, we concluded that ABA, auxin and SL were involved in regulation of axillary bud outgrowth in response to low R:FR. In addition, we also inferred that low R:FR caused the increase of ABA content and decrease of sucrose accumulation in upper buds which suppressed the upper bud release, on the contrary, sucrose accumulation in bottom buds promoted the bottom bud release and facilitated the bud outgrowth finally. Therefore, ABA and sucrose may assume important responsibilities for divergent bud outgrowth of upper and bottom buds in response to low R:FR. Of course, further evidences are needed to support our speculation. Despite all this, our study opens a window for considering bud position and sucrose in future similar studies. The present study also lays the foundation for using R:FR to regulate axillary but outgrowth.

## 4. Materials and Methods 

### 4.1. Plant Materials and Growth Conditions

Single-flower cut Chrysanthemum ((*Dendranthema grandiflorum* ‘Jinba’)) cuttings of uniform length, containing at least two buds, were obtained from healthy stems using sharp secateurs. Cuttings were grown in 50H-Cutting tray Drip trays with 50 cuttings in each tray in the greenhouse of Beijing Forestry University. After 20 days, the health seedlings were selected and shifted to 12 cm × 12 cm nutrition cups containing mixture of nutrient soil and vermiculite (2:1 ratio). Then, the seedlings were used for experiment. The following two light environments were designed in the artificial climate chamber: (1) Normal lighting environment (High R:FR, W): the use of 4 Philips T8 TLD36/33 cold white tubes, providing 120 μmol m^−2^ s^−1^ PPFD; (2) Simulated shade environment (Low R:FR, W + FR): in the normal lighting environment added two sets of 735-nm LEDs far red tubes to provide supplemental far-red light and to decrease the R:FR without altering the PPFD. Both high- and low-R:FR conditions were maintained in the same artificial climate chamber using a barrier to prevent light interference with each other. The photoperiod and temperatures in the growth chamber were 16 h-light/8 h-dark and 24 °C-day/18 °C-night, respectively.

To investigate whether treatment period, way and time will affect the fate of Chrysanthemum axillary buds, the following treatments were explored:

(1) Plants were grown in low R:FR throughout the development stage (Low R:FR).
(2) Plants were grown in high R:FR throughout the development stage (High R:FR).
(3) Plants were first grown in low R:FR for 10 days at early seedling stage, then grown in high R:FR (Low R:FR 10 days + High R:FR).
(4) Plants were first grown in high R:FR for 41 days (nearly 45 cm height), then either maintained under high R:FR or provided with low R:FR (High R:FR 45 + Low R:FR).
(5) Plants were first grown in low R:FR for 41 days (nearly 45 cm height), then either maintained under low R:FR or provided with high R:FR (Low R:FR 45 + High R:FR).
(6) Plants were first grown in high R:FR for 64 days (nearly 60 cm height), then either maintained under high R:FR or provided with low R:FR (High R:FR 60 + Low R:FR).
(7) Plants were first grown in high R:FR for 41 days (nearly 45 cm height), then 1/2 plants were maintained in high R:FR and another 1/2 provided with low R:FR for 12 days, then re-grown in high R:FR (High R:FR 45 + Low R:FR 12 days + High R:FR).
(8) Plants were first grown in low R:FR for 41 days (nearly 45 cm height), then 1/2 plants were maintained in low R:FR and another 1/2 provided with high R:FR for 12 days, then re-grown in low R:FR (Low R:FR 45 + High R:FR 12 days + Low R:FR).

The length of axillary buds was measured using digital Vernier calipers. Data were recorded for 15 plants per treatment with 3 replicates.

### 4.2. Determination of Hormone Content

Keeping in view the Chen’s [47] findings combined with our observation that Chrysanthemum ‘Jinba’ varieties generally begin to axillary bud initiation at a height of 45 cm, the plants were first grown in high R:FR (W) before 45 cm height, then half of the plants (nearly 45 cm height) were still maintained under high R:FR, and another half were provided with low R:FR (W + FR). After 12 h, tip buds, upper axillary buds and bottom axillary buds based on their location in the stem were sampled for hormone examination. The samples were stored in −80 °C. Then the GA, ABA, IAA and CK (ZR) contents were measured by enzyme-linked immunosorbent assay (ELISA). Data were recorded for 15 plants per sample with 3 replicates. 

### 4.3. Determination of Sucrose Content

The treatment strategy was the same as the hormone content determination. Tip buds, upper axillary buds and bottom axillary buds were sampled after 2 h, 6 h, 12 h and 24 h. The samples were stored in −80 °C. Sucrose concentration was determined following the method of Wang [76] with little modification. Briefly, bud samples were ground in liquid nitrogen and extracted thrice in 80% ethanol. The supernatant were pooled and filtered in carbon black and final volume was made to 25 mL by adding distilled water. Reaction mixture contained 900 µL extract and 100 µL of 2 N NaOH followed by boiling for 10 min in water bath at 80 °C. Then, 3 mL of 10 N HCl and 1 mL of 0.1% resorcinol were added and boiled at 99 °C for 1 h. After that absorbance was checked at 480 nm using a UV-1700 PharmaSpec spectrophotometer (Shimadzu Corporation, Kyoto, Japan) with 20 µg/mL sucrose solution as standard. Standard curve was obtained using 20 µg/mL sucrose concentration with an acceptable correlation coefficient (R^2^ = 0.998) (Figure 3b).

### 4.4. Quantitative Real-Time RT-PCR Analysis

The treatment strategy was the same as the hormone content determination. Upper axillary buds and bottom axillary buds were sampled after 8 h. The samples were stored in −80 °C until RNA extraction. Total RNA was extracted from frozen buds using MiniBEST plant RNA extraction kit (TaKaRa Bio Inc., Dalian, China) including DNA removal as well, finally obtaining DNA-free RNA. cDNA was obtained by performing reverse transcription of 1 µg RNA using PrimeScriptTMRT reagent kit/system (TaKaRa Bio Inc., Dalian, China). Quantitative real-time PCR (qRT-PCR) experiments were carried out with TaKaRaSYBR^®^Premix Ex Taq TMΠ (Tli RNaseH Plus) (TaKaRa Bio Inc., Dalian, China) using a total mix of 15 µL including: 7.5 µL SYBR^®^Premix Ex Taq TMΠ (Tli RNaseH Plus) (2×), 0.6 µL forward primer (10 µM), 0.6 µL reverse primer (10 µM), 1.2 µL cDNA, 5.1 µL dH_2_O. CFX Connect Real-Time System was set to following programme: 30 s at 95 °C, then 40 cycles of 5 s at 95 °C and 30 s at 60 °C. Specific primers used for qRT-PCR are shown in Supplementary Materials Table S1. Each qRT-PCR reaction was done in triplicate on three independent biological samples. Relative gene expressions were quantified using the expression of *DgActin* as internal control.

### 4.5. ABA Treatments

The split-plate system was conducted according to previous report. This assay have been used in previous studies to examine the effect of exogenous hormones on bud outgrowth in Chrysanthemum [47]. MS media supplemented with DMSO, 1 μm ABA, 10 μm ABA, 100 μm ABA, 10 μm ABA biosynthesis inhibitor fluridon, and 100 μm ABA + 10 μm fluridon was made. A split gap was made in the middle of the plate, then a single bud-node section was inserted in the split-plate. The bud length was measured every day by using camera (Nikon D7000, Tokyo, Japan) and ImageJ software (ImageJ IJ1.46r, National Institutes of Health, Bethesda, MD, USA). 24 buds were used for each treatment with 3 repeats.

### 4.6. Statistical Analyses

The One-way ANOVA analysis was done using the SPSS software (ver. 16.0; SPSS Inc., Chicago, IL, USA). Statistically significant differences were indicated at *p* < 0.05 or *p* < 0.01 level.

## Supplementary Materials

Supplementary materials can be found at http://www.mdpi.com/1422-0067/19/6/1590/s1.

## Abbreviations

R:FR	Red light to far-red light ratio
ABA	Abscisic acid
SL	Strigolactones
CK	Cytokinin
IAA	Indole-3-acetic acid
GA	Gibberellin
MAX	More axillary branching
PAT	Polar auxin transport
PIN1	PIN-FORMED 1
phyB	Phytochrome B
SAS	Shade avoidance syndrome
CCD	Carotenoid cleavage dioxygenase
