# Supplementary material for: Red to Far-Red Light Ratio Modulates Hormonal and Genetic Control of Axillary bud Outgrowth in Chrysanthemum (Dendranthema grandiflorum ‘Jinba’)
Source: Int J Mol Sci. 2018 May 28;19(6):1590. doi: 10.3390/ijms19061590 (PMC6032274; doi:10.3390/ijms19061590)
Supplement: Supplementary file 1 [file ijms-19-01590-s001.pdf]

International Journal of
Molecular Sciences

MDPI

Table S1. Primers used for gene expression analyses by qRT-PCR.

Gene Name	Primer Sequence (5′-3′)	Gene Name	Primer Sequence (5′-3′)
DgBRC1	F:CCCTTTTGGAGAGCATCAAG	*DgPIN1*	F:TGGCATTGCAACCAAGGATC
	R:AGACGTCGCGGATGAAGTAT		R:AAATGGAAGCAGCAGCCATG
DgMAX1	F:GAGTTGAAGTTGGAGGTTACG	*DgABA2*	F:TGGGATACTGCGCTTTTCGT
	R:GACCAAGGCACATTCGAGG		R:ACCGGAACATAGCCAACACG
DgCCD7	F:TGGTGAAGTTCGATACTGTG	*DgNCED2*	F:TCGATGTTGGGCTTGGTTGA
	R:CGTCGCTACCCTTTGATAC		R:CGGGTGAGCTATCATCGTGG
DgCCD8	F:CATTGTTGCCCGATCTGTT	*DgABF3*	F:GAATTATGGCCGGGTCTCCA
	R:GGCCAATCACCTTCCTCT		R:CTCCGCCTTCTCTCCACAAC
DgMAX2	F:GCACATACTGCACCATC	*DgABF2*	F:TGAGGGGTAGGAGAACTGGT
	R:GTAACGACAAACTCCTCTGG		R:GCCTGTTTACGAGCCCTTGA
DgCYP79B2	F:TACACGGCAATCCCCAAGAG	*DgABIL1*	F:ATGGTGAAGAGGGAATGCCG
	R:GTTTAGCACCGGGAAACGTG		R:TCGTGTGATGCCAAGTGTCT
DgCYP79A1	F:TCCGTCTCGTTTTGGTTGGA	*DgHAB1*	F:TGGGCATCGTGTTTTTGGTG
	R:TCAGCCGTCATGTCTCCTTT		R:CATCACGTCCCATAACCCGT
DgIAA16	F:CCTTCACCATCGGTAGCTGT	*DgPDS1*	F:ACCGGCATCAGCCAAATACT
	R:CGACGAGCATCCAATCTCCA		R:TTGACTATCCACGCCCTGAC
DgIAA9	F:TGGTGACTGGATGCTTGTGG	*DgRD19A*	F:TGAGGTTCAAGGACAAGCCA
	R:GCTTTCCGACCAAACAGTGG		R:GGTGTCCATCCCACAAGCAT
DgARF2	F:AAACCTTTGCTCTGGTGCCT	*DgACTIN*	F:GACTGATGCGTTGATGAAGA
	R:CTGCGGAGTTCATCGTTCCT		R:TCATGAATACCAGCAGCT
DgGH3.5	F:ATGCCGGTGGAGTACCTTTG		
	R:CGGAAGCACAGCGAAAGTTG		
